# Mitochondrial DNA mutations in Parkinson's disease brain

**DOI:** 10.1186/s40478-017-0433-9

**Published:** 2017-04-29

**Authors:** David K. Simon, Joanne Clark Matott, Janaina Espinosa, Neeta A. Abraham

**Affiliations:** Beth Israel Deaconess Medical Center, Harvard Medical School, 330 Brookline Ave. Room CLS-638, Boston, MA 02215 USA

## Please see the related Research article (10.1186/s40478-016-0404-6) and the related Letter to the Editor (10.1186/s40478-017-0434-8)

Dear Editors,

We read with interest the publication by Wei et al., Mitochondrial DNA Point Mutations and Relative Copy Number in 1363 Disease and Control Human Brains, *Acta Neuropathol Commun*. 2017; 5: 13 [4]. We disagree with the authors’ conclusion that their data indicate that, “single nucleotide variants of mtDNA are unlikely to play a major role in the pathogenesis of these neurodegenerative disease.” There are 4 specific reasons why we disagree with this conclusion, and instead we assert that their data are not contradictory to our own previously published data indicating significantly elevated levels of heteroplasmic mtDNA mutations in dopaminergic neurons in the substantia nigra (SN) of Parkinson’s disease (PD) patients compared to controls at very early pathological stages of PD [2]. 1) Wei et al. looked at cerebellar tissue for 1189 out of the 1363 cases, and so their data do not address the levels of mtDNA mutations within a pathologically affected tissue in PD. In contrast, in our prior publication we looked specifically at the SN; 2) Wei et al. looked at brain homogenate rather than specifically at vulnerable neurons. In contrast, in our prior publication we looked specifically at dopaminergic neurons isolated by laser capture microdissection (LCM); 3) Wei et al. did not discuss the pathological stage of the disease for the brain tissue that was studied. In most cases, postmortem brain tissue available from PD patients is at an advanced pathological stage at which point most dopaminergic SN neurons have died. In contrast, in our prior publication we analyzed SN neurons from early pathological stages of PD separately from tissue demonstrating advanced pathological stages. 4) Wei et al. defined mutations as being homoplasmic if they were present in <10% or >90% of reads. However, our prior studies found numerous point mutations in mtDNA that were individually rare (present in <10% of mtDNA copies), but that, in aggregate, reach a high mutational burden. In agreement with Wei et al., we previously reported no differences in heteroplasmic mtDNA point mutations in PD compared to controls when analyzing late-stage brain homogenate (SN in the case of our study) [3]. We hypothesized that mutations might preferentially accumulate in neurons rather than other cell types, which we later confirmed [1], and that those neurons that accumulate high levels of mutations die, and so are not present for analysis in end-stage tissue. This led to our subsequent study using LCM to analyze heteroplasmic mtDNA point mutation levels in early-stage dopaminergic SN neurons, which revealed significantly higher levels of mutations compared to levels in either age-matched controls or in advanced stage PD SN neurons. Therefore, in contrast to their conclusion, the data presented in Wei et al. does not contradict our prior work, and does not provide an argument against our hypothesis that somatic mtDNA point mutations accumulate within dopaminergic SN neurons and contribute to the degenerative process in PD.

Sincerely,

David K. Simon, MD PhD
